# Origin of Cancer: An Information, Energy, and Matter Disease

**DOI:** 10.3389/fcell.2016.00121

**Published:** 2016-11-17

**Authors:** Rainer G. Hanselmann, Cornelius Welter

**Affiliations:** ^1^Institute of Human Genetics, Saarland UniversityHomburg, Germany; ^2^Beratungszentrum für HygieneFreiburg, Germany

**Keywords:** entropy, cancer, carcinogenesis, warburg effect, mutations, aneuploidy, microenvironment, matter

## Abstract

Cells are open, highly ordered systems that are far away from equilibrium. For this reason, the first function of any cell is to prevent the permanent threat of disintegration that is described by thermodynamic laws and to preserve highly ordered cell characteristics such as structures, the cell cycle, or metabolism. In this context, three basic categories play a central role: energy, information, and matter. Each of these three categories is equally important to the cell and they are reciprocally dependent. We therefore suggest that energy loss (e.g., through impaired mitochondria) or disturbance of information (e.g., through mutations or aneuploidy) or changes in the composition or distribution of matter (e.g., through micro-environmental changes or toxic agents) can irreversibly disturb molecular mechanisms, leading to increased local entropy of cellular functions and structures. In terms of physics, changes to these normally highly ordered reaction probabilities lead to a state that is irreversibly biologically imbalanced, but that is thermodynamically more stable. This primary change—independent of the initiator—now provokes and drives a complex interplay between the availability of energy, the composition, and distribution of matter and increasing information disturbance that is dependent upon reactions that try to overcome or stabilize this intracellular, irreversible disorder described by entropy. Because a return to the original ordered state is not possible for thermodynamic reasons, the cells either die or else they persist in a metastable state. In the latter case, they enter into a self-driven adaptive and evolutionary process that generates a progression of disordered cells and that results in a broad spectrum of progeny with different characteristics. Possibly, 1 day, one of these cells will show an autonomous and aggressive behavior—it will be a cancer cell.

## Introduction

Since 1858, when Rudolf Virchow formulated the idea that cancer cells are the body's own cells (Virchow, 1858), many hypotheses have been proposed to explain the origin of cancer cells and how they develop such a heterogenetic morphology, increased proliferation, metastatic capacity, and invasive behavior.

Several theories have been formulated, but even the most prominent and accepted model (mutation theory) are confronted by a growing amount of experimental data and arguments that could either not explained by the model, or that contradicted this model. For example, Mack et al studied subtypes of three ependymoma brain tumors and found that one subtype carries an intrachromosomal translocation that creates a new tumor-driving gene, another lacks tumor-driving mutations but has aberrant epigenetic modifications, and a third shows neither gene mutations nor epigenetic aberrations (Mack et al., 2014; Versteeg, 2014). On the other hand, Martincorena and colleagues found thousands of mutations in cancer-relevant genes, including cancer-driver genes, in normal eyelid epidermis that rarely develop cancers (Martincorena et al., 2015). This finding stands in contrast to the mutation hypothesis because, if mutations really are the exclusive cause of cancer, then how can the hypothesis explain, on the one hand, the existence of cancers *without mutations*, and on the other, the fact that *normal* tissues can display massive genetic changes including changes in cancer-initiating and cancer-driving genes? Furthermore, during recent decades, in several transfer experiments (nucleus and mitochondrial transfer) the tumor-suppressing effect of normal cytoplasm, as well as of normal mitochondria, could be demonstrated, despite the presence of cancerous nuclear genomes (Seyfried, 2015). For example, Kaipparettu et al. (2013) were able to show that the introduction of non-cancerous mitochondria into highly malignant breast cancer cells could reverse malignancy and down-regulate several oncogenic pathways such as invasion, *in vivo* tumor growth, and others. Moreover, there are several non-genotoxic (non-mutagenic) carcinogens including chloroform and *p*-dichlorobenzene that provoke cancer formation (Mally and Chipman, 2002; Duesberg et al., 2011; Seyfried, 2015). However, we saw in the last decade the renaissance of old (Hansemann, 1892; Boveri, 1914; Duesberg, 2005; Duesberg et al., 2011; Seyfried, 2015) and the formulation of new hypotheses (Baker, 2015; Tomasetti and Vogelstein, 2015) that have all failed to show that they are the exclusive initiator and origin of cancer. For example, Warburg (1956) suggested that the disturbance of oxidative phosphorylation which results in cellular energy loss has a high impact on cancer formation, though this concept cannot explain the fact that hereditary mitochondrial diseases are not associated with an increased rate of cancer formation, even when most mitochondria are affected (Moggia et al., 2014). On the other hand, in recent years, several studies have shown a causal link between oncogenic genes, and the disturbance of mitochondria. For example, Matoba et al. (2006) observed that p53^−/−^ mice exhibit impaired mitochondrial respiration, and Bensaad et al. (2006) identified a novel p53-inducible regulator of glycolysis. Actually, there is a controversial debate as to whether mutated cancer-relevant genes or disturbed mitochondria are the initial cause of cancer formation. In that discussion, we have to take into account that, besides mitochondria, mutated oncogenes such as that encoding E2F transcription factor (Benevolenskaya and Frolov, 2015) or p53 (Kamp et al., 2016) simultaneously affect other cellular mechanisms such as DNA repair and mitosis (Vitale et al., 2010). For that reason, it will be difficult to decide which is the primary cause and whether either is responsible exclusively for cancer initiation.

At the beginning of the last century, another popular hypothesis was published concerning the origin of cancer. It was formulated by Theodore Boveri, a German biologist (Boveri, 1914). Based on his own experiments with sea urchin eggs and the works of (Hansemann, 1892), he formulated a comprehensive monograph about the origin and possible causes that he supposed would lead to chromosomal imbalance in post-mitotic daughter cells (aneuploidy) and finally to cancer. Whereas, Boveri principally assumed that single mitotic errors can lead to cancer cells, most scientists today believe that once aneuploid cells occur, a process of chromosomal instability (CIN) becomes initiated, which is the basis for cancer cell formation through an evolutionary process. This model implies that aneuploidy itself destabilizes karyotypes automatically and impels progression toward CIN and cancer (Duesberg, 2014) on its own terms.

Today, it is clear that aneuploidy is a common genetic feature of solid tumors (Hanahan and Weinberg, 2011). Whether mutation in cancer-related genes (Holland and Cleveland, 2009; Thompson et al., 2010; Holland and Cleveland, 2012), or accidental appearance of chromosome mis-segregation during mitosis (Thompson et al., 2010; Duesberg et al., 2011), or by tetraploid progenitor cells (Ganem et al., 2007; Storchova and Kuffer, 2008), or because of epigenetic mechanisms (Herrera et al., 2008), or centrosome aberrations (Nigg, 2002) or other possible causes is under intensive investigation and discussion. However, there is some evidence indicating that aneuploidy cannot be the only reason for carcinogenesis. For example, Weaver and Cleveland and others found that aneuploidy suppresses rather than promotes tumorgenesis (Weaver and Cleveland, 2007; Weaver et al., 2007; Torres et al., 2008), but if aneuploidy is so deleterious, why, then, are most solid tumors aneuploid? Furthermore, individuals with Down syndrome carrying an extra copy of chromosome 21 have a 50% lower risk of developing a solid tumor but a significantly higher risk of developing leukemia, as compared to individuals with a normal karyogram (Hasle et al., 2000). On the other hand, aneuploidy is an early event found in typical pre-cancer stages such as cervix dysplasia, Barrett esophagus, leukoplakia and bronchus dysplasia (Sandritter, 1965; Reid et al., 2000; Hanselmann and Oberringer, 2001; Lothschütz et al., 2002; van Zyl et al., 2012) liver cirrhosis (Attallaha et al., 1999) and others (Bohm and Sandritter, 1975). It is interesting that aneuploidy is even detectable in chronic inflammatory tissue such as wounds (Ermis et al., 1998; Oberringer et al., 1999) and inflammatory bronchial tissue (Hanselmann and Oberringer, 2001; Lothschütz et al., 2002), after acute and chronic hypoxic conditions (Ueyama et al., 2012; Kondoh et al., 2013), and after exposure to physical (Grosovsky et al., 1996; Kirsch-Volders et al., 1996) or chemical stressors (Galloway and Ivett, 1986; Mattiuzzo et al., 2006; Tayama et al., 2008). However, there are many pros and cons in the context of aneuploidy as a reason for cancer development, but as Yuen and Desai assume, the persistence of aneuploid cells in tumors requires not only chromosome mis-segregation but also additional, as yet poorly defined, events (Yuen and Desai, 2008).

In summary, at the time of writing, there is no experiment that proves that any of the hypotheses that have been propounded represents the exclusive cause of cancer. For further arguments, please see Table 1.

**Table 1:** **Pros and cons of different theories and hypotheses indicate that no one theory can claim to be the exclusive cause of cancer initiation and progression**.

**Theory, hypothesis**	**Pros**	**Cons**
Mutation theory	Mutations are detectable in most cancers (Hanahan and Weinberg, 2011). Hereditary cancer diseases (Hanahan and Weinberg, 2011). Induction of cancer by targeted mutation of cancer genes in animals (Ko et al., 2004). Mutations can induce aneuploidy and CIN (Lengauer et al., 1998).	Normal tissue shows high incidence of mutations even in cancer-driver genes (Martincorena et al., 2015). Physiological mutation rate cannot explain cancer development (Duesberg, 2005; Duesberg et al., 2011). Identification of cancer disease without cancer-relevant mutations (Mack et al., 2014; Versteeg (2014). Mutations cannot explain the pathogenetic causality of early mutation appearance and years of latency of cancer development (Duesberg, 2005; Duesberg et al., 2011). Cannot explain mitochondrial and nucleus transfer experiments (see: Introduction, Seyfried, 2015).
Chromosomal imbalance, Speciation theory, Aneuploidy and CIN	Aneuploidy is detectable in most tumors (Duesberg et al., 2011). Aneuploidy is detectable in pre-cancer stages [Barrett esophagus (Reid et al., 2000), chronic infection (Attallaha et al., 1999); chronic inflammation (Lothschütz et al., 2002)].	Not every cell within a tumor shows chromosomal aberrations (Lengauer et al., 1998). Constitutional aneuploidy in the normal human brain (Rehen et al., 2005). Cannot explain mitochondrial and nucleus transfer experiments (see: Introduction, Seyfried, 2015).
Mitochondrial dysfunction, Energy loss (Warburg effect)	Detectable in all tumors (Seyfried, 2015). Induction of cancer by transfer of dysfunctional mitochondria (Seyfried, 2015). Induction of cancer by mitochondrial toxic agents (Warburg, 1956).	Mitochondrial dysfunctions are not the initial step in hereditary cancer development (see introduction). No hereditary mitochondropathies show an increased cancer rate (Moggia et al., 2014). The hypothesis cannot explain the development of cancer by many substances that directly disrupt mitotic spindle aperture and induce aneuploidy and CIN (Duesberg, 2005; Duesberg et al., 2011).
Environment and matter	Micro-environmental changes can induce cancer (Gatenby and Gillies, 2008; Baker, 2015) such as hormones (Tsutsui and Barrett, 1997), low pH (Takeshi, 1995).	Micro-environmental changes are not the first step in the development of hereditary cancers, because inherited mutations are the initiator of that process (Hanahan and Weinberg, 2011; Tomasetti and Vogelstein, 2015).

Furthermore, when we are looking at a broad range of hypotheses that show, experimentally, a varying degree of impact on cancer initiation and progression, it is difficult to believe that any single model can lay claim to explaining the cause and progression of cancer diseases exclusively. To overcome the dilemmas of correct-in-principle, but competing models, the establishment of an overarching framework that combines these different models is reasonable. In order to establish such a framework, it makes sense to identify the higher principles that lie behind each individual cancer model, creating the opportunity to place them within a small number of basic categories and to bring them into the context of one of the most fundamental laws of nature—thermodynamics.

## Basic principles of life

For more than 2000 years, humans have tried to define the basic principles of the universe (Umpleby, 2007), leading us to fundamental theories such as quantum mechanics and Albert Einstein's general theory of relativity. Based on those fundamental theories, general systems theorists emphasized that matter, energy and information are basic categories in our universe that are linked at the quantum level, and for that reason, some assume that they must be fundamental for life too (Miller, 1978; Umpleby, 2007).

A cell is the fundamental unit of life: capable of self-preservation by self-organization and autonomous reproduction (Karsenti, 2008; Davies et al., 2013). In recent decades, it has become clear that a cell is a thermodynamically open system (Alberts et al., 2011) that is far from thermodynamic equilibrium, and it is generally accepted that such systems need a permanent supply of energy for self-preservation (Alberts et al., 2011; Molnar et al., 2011; Davies et al., 2013; Gatenby and Frieden, 2013; Tarabichi et al., 2013). For that reason, cells permanently ingest fuels (e.g., glucose, oxygen) and generate energy-bearing molecules (e.g., ATP, GTP) mainly by respiration (Alberts et al., 2011). Energy is vital for the cell and it is involved in all molecular biological and biochemical processes. This dependency becomes obvious when cells completely disintegrate structurally and metabolically after they have lost too much energy (cell death = entropy). But to preserve the steady state of order, the cell must ingest nutrition (matter) such as amino acids, lipids, carbohydrates or electrolytes from the surrounding environment (Alberts et al., 2011). Under physiological conditions, the cell's surroundings are perfectly adjusted to the cell's needs, but what happens when the environment changes significantly or the nutrition supply becomes disturbed? In the past, many environmental changes such as hypoxia, chronic inflammation, irradiation and hormones have demonstrated that they can induce or support cancer cell formation (Gatenby and Gillies, 2008; Rademakers et al., 2008; Tayama et al., 2008; Castello et al., 2010; Nishisgori, 2015; Michiels et al., 2016), and from studies of cancers it is well known that they are surrounded by massive environmental changes (Gatenby and Gillies, 2008; Hanahan and Weinberg, 2011; Gillies and Gatenby, 2015). These findings clearly indicate that environmental (matter) changes have a great impact on cancer development. However, without genetic information, neither the construction nor the preservation of life is possible. Genes and their resulting products guarantee that the intricacy of cellular order (e.g., structures, defined reaction spaces (organelles) and reaction cascades) is preserved and transferred from generation to generation (Alberts et al., 2011). Supporters of the mutation and the aneuploidy hypotheses in particular emphasize the importance of the genome for life and focus their efforts on that single component in order to explain carcinogenesis.

**Figure 1:**
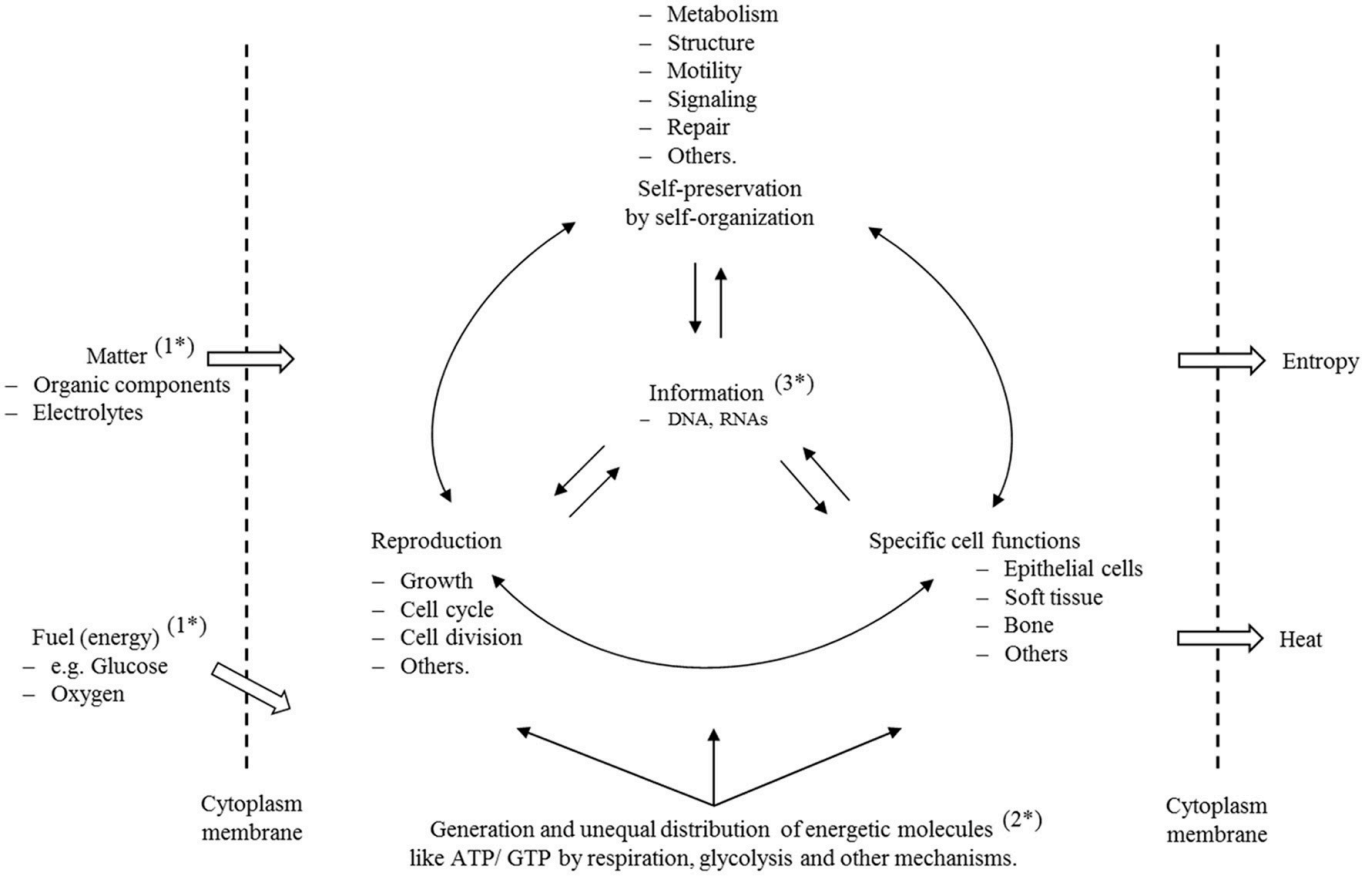
**Self-preservation and reproduction are the fundamental characteristics of cells**. To preserve all structures and functionalities, the cell must ingest matter (1^*^) to generate structural and functional, as well as specific energetic, molecules (2^*^). To meet these highly sophisticated properties, life has developed, through an evolutionary process, a molecular information system (3^*^) that guarantees structure, ordered processes, and a safe transfer of information from generation to generation. The scheme illustrates that matter, energy and information depend reciprocally on each other and, on this basis, it is clear that disturbance of any one of those properties must have consequences for the others. A cell is a thermodynamic, open system that is far away from equilibrium. For this reason, it displays a lower entropy state than the environment. Furthermore, we have to assume that entropy is not equally distributed, and that the complete entropy of a cell is in fact a sum of many different individual states. Disturbance of individual molecular mechanisms and structures has different consequences to the cell, but, if they are important enough and reach a state of irreversibility that cannot be repaired or compensated, then the cell enters a metastable state with all of the consequences that are explained in this text.

In Figure 1, we illustrate in a simple scheme the importance of the complex interplay and reciprocal dependency of these three basic categories (matter, energy and information) and we invite the reader to investigate this fundamental interplay and to decide afterwards whether these categories are equally important to preserve life and order, or whether any one category can claim this right exclusively.

If we accept that matter, energy and information are the basic categories that underpin and permit life, then a question emerges in the context of cancer: how can we assume that only the disturbance of information, through mutations, is exclusively able to initiate, and afterwards, drive, a process that is finally characterized by a chaotic progression of genomic, metabolic, morphological and other cell characteristics?

We suggest that in principle any perturbation of information, energy or matter is able to initiate undefined molecular changes within the ordered cell system that is described by the state function, entropy. This first event appears locally or generally, depending on the type and dimension of the affected basic category, and initiates and promotes a chaotic, self-organizing, and to some extent stochastic, process that can finally generate a cancer cell. In the following chapters, we will describe the impact of information, energy and matter on that process and discuss their importance in the initiation of increased entropy and disorder, and how they interact to promote cancer formation. But first of all, we would like to focus on that state function which describes the mechanisms responsible for cancer initiation and progression—entropy.

## Entropy and irreversible disorder

The second law of thermodynamics is the law that describes entropy. Entropy is a state function and a dimension for the increase in reaction probabilities, irreversibility, chaos and disorder in an ongoing system (Alberts et al., 2011; Davies et al., 2013; Tarabichi et al., 2013; Gomez, 2014). The law says that in a closed system entropy either stays the same or gets bigger, and it therefore predicts that the universe tends toward maximum entropy (Wolfe, 2015). For that reason, entropy defines a direction for all processes and reactions. A cell, however, is an enveloped but open thermodynamic system. That means that a cell has the opportunity to exchange both matter and energy with an outside system (the extracellular environment) and can release degradation products (positive entropy) such as CO_2_, H_2_O, heat and others (described here as thermodynamic waste). Because of that capability, the cell is able to maintain and increase cellular order within a progressively entropic environment. It therefore controls and provides optimal reaction conditions to generate structures and to preserve reaction cascades in circumscribed spaces. Principally, we can regard all of these intracellular mechanisms and structures as many single reaction states that become influenced by their intracellular local environment. For example, the structural order and the functionality of proteins are highly dependent upon conditions such as pH, electrolyte concentration and temperature (Wirth et al., 2013; Uversky, 2015). Under physiological conditions, the cell is able to control reaction probabilities in these different intracellular microenvironments and therefore it needs genomic information, nutrition (matter) and fuels (energy) (Alberts et al., 2011; Tarabichi et al., 2013; Gomez, 2014;). All of these processes are highly dynamic and the cell consumes a huge amount of energy to sustain all functionalities (Davies et al., 2013).

**Figure 2:**
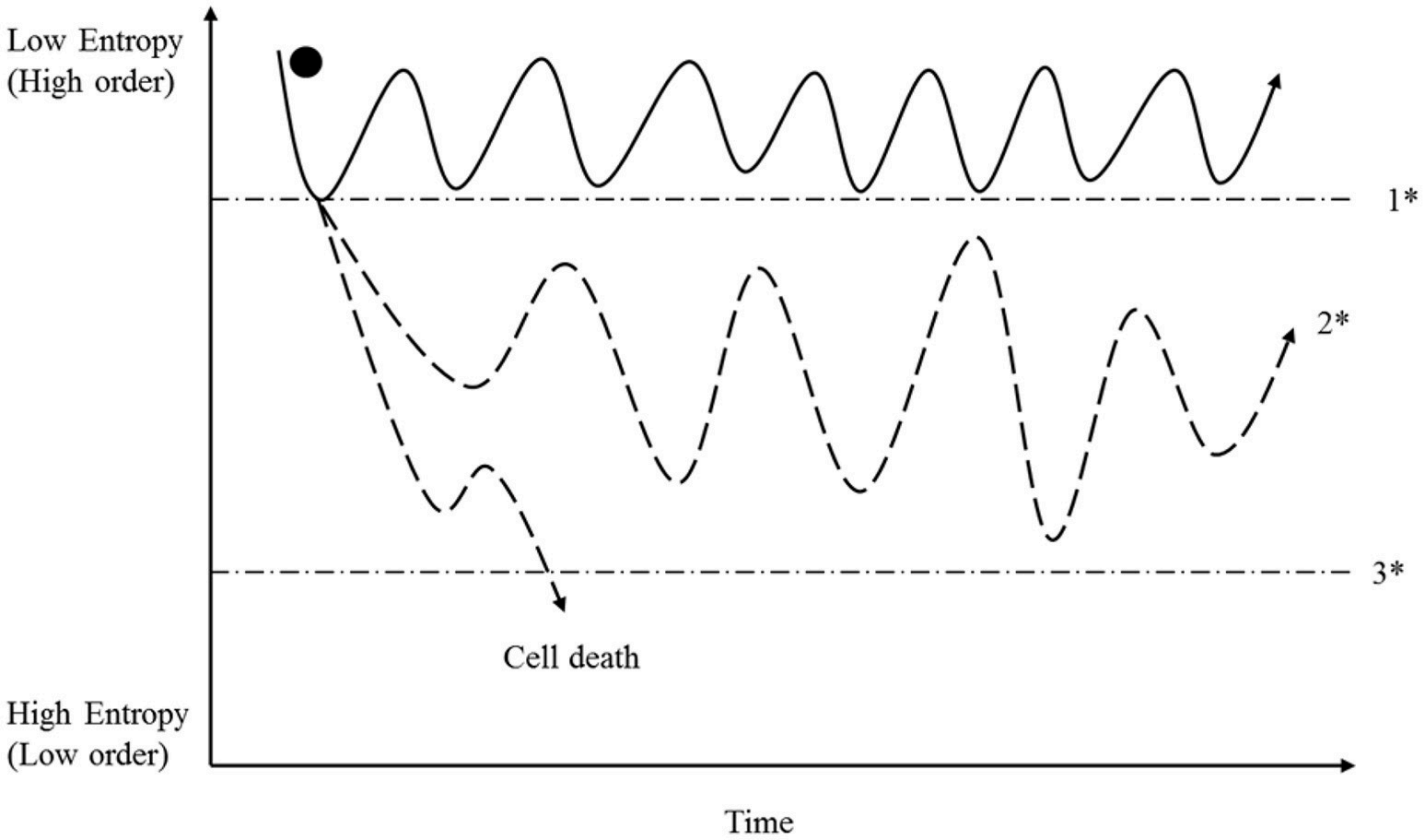
**A cell has the opportunity to sustain high order and self-preservation by utilizing energy and matter; and organization is achieved through the use of information molecules (see Figure 1)**. For reasons provided in the preceding text, entropy can fluctuate reversibly within a specific range (area above 1^*^). In that context, we note especially that the described entropy fluctuation is a result of many individual entropic states representing reactions, mechanisms and structure. If one or more fluctuations exceeds a reaction-specific breaking point (1^*^), irreversibility sets in, and the future of the cell then depends upon its capability to stabilize itself in a metastable state (2^*^). This capability depends on adequate information (Figure 3), energy availability (Figure 4) and the supply of matter needed. If the degree of disorder is too strong, then cells pass the breaking point (3^*^) and die. In contrast, metastable cells persist in a process of permanent adaptation including the capability to reduce entropy locally (increase order) that can finally lead to cancer cell formation, as described in the main text. It is important to note that energy, matter and information are able individually to provoke the passage through the breaking point (1^*^); this is described in the main text, and in Figures 3, 4.

However, based on the law of thermodynamics, we can deduce the following for highly complex, open thermodynamic systems such as cells: in reversible processes, entropy fluctuates in a defined dimension, whereas in irreversible ones, entropy has exceeded a point of no return that makes the reversal of the initiated changes impossible (see Figure 2). These fundamental perceptions are of central importance when we try to understand the origin and drivers of cancer (see below).

In physics, a stable state of a system is an equilibrium state (Wolfe, 2015). When a process reaches equilibrium, reactivity stops because all driving forces are weakened. All systems follow this basic rule. However, because of its high degree of order, a cell is far away from equilibrium. This is possible because cells are open thermodynamic systems, which gives them the opportunity to permanently ingest matter and energy and to release waste, and thereby to preserve order (low entropy). Moreover, during evolution, the cell has gained a set of information molecules (e.g., DNA, RNA, and proteins) that guarantees self-preservation by self-organized processes and autonomous reproduction, and the transfer of that information from generation to generation (Alberts et al., 2011).

A consequence of the above is that the preservation of order (low entropy) in a cell depends upon these fundamental categories: information, energy and matter. If one of these categories is seriously disturbed, then cellular entropy increases (structurally or functionally), and an irreversible disorder may become established (Figure 2). What opportunities does a cell have in such a threatening situation? We postulate that at this stage a process is initiated that drives molecular mechanisms that lead to further increased intracellular entropy and that, in opposition to this process, competing cell reactions try to stabilize metabolism and cell integrity. The driving conflict behind these self-promoting mechanisms is that cells try to manage locally increased entropy through the interplay of locally available information, energy and matter. Because the second law of thermodynamics cannot allow for reversal of irreversible disorders, the cell is forced to establish a new order, i.e., to find a new stable state. A characteristic of this adaptive process is that regular cell processes reach points of progression where they come into conflict with irreversible disordered molecular mechanisms. The cell now has various options: first, if the disorder state is not “strong or relevant” enough, then it becomes compensated by the cell; second, if disorder is too strong, then the affected cell dies; or third, if the initiating event leads to a metastable state that is principally compatible with life, then it represents the base that maintains cell life, but at the same time it is the pre-condition for the progress of the molecular disorder that can finally lead to cancer cell formation (Figure 2).

The principle of metastable cells is of central importance to our hypothesis. For that reason, it is important to explain what metastability means. As already mentioned, normal cells are highly ordered and preserve cell integrity and proper behavior within a functional tissue. They guarantee the safe transfer of cellular order from generation to generation. If that sophisticated and precisely orchestrated interplay becomes irreversibly disturbed (locally or generally), then the cell will never be the same because, in that moment, a functional or structural change becomes established within the cell that it can neither control nor repair. This change will constantly disrupt dependent mechanisms and, if disorder increases as a result, then the primarily ordered system becomes more and more chaotic. However, the cell loses efficiency and if that change provokes further disorder, then efficiency will decrease simultaneously. The decrease of efficiency must be associated with an increased release of heat and, in fact, tumor cells are characterized by an increased production and release of heat (Molnar et al., 2011). The cell has the opportunity to compensate for this situation through various mechanisms (see below) and it is this interplay of persisting or increasing disorder, alongside cellular reactions that try to preserve life, which characterizes metastability.

In this context, several aspects are important. First, metastability is not based on predetermined events that lead to cancer; it is based on reaction probabilities outside of cellular control, and because of these probabilities, cell mechanisms and structure change to some extent stochastically until local or general equilibrium becomes established. The cell tries to compensate for this threat by activating various mechanisms including stress responses, energetic adaptation, apoptosis and epigenetic mechanisms and the cell changes continuously based on this sophisticated interplay. We believe that this is one of the reasons why pre-cancer cells and cancer cells display such significant metabolic changes (Cairns et al., 2011; Keenan and Chi, 2015) and an amazing morphological and genetic heterogeneity and behavior during progression (Hanahan and Weinberg, 2011).

The second important aspect is that the time period between the perturbation of the first category (information, energy or matter, which induces the process) and the involvement of the remaining categories is not clear and has to be evaluated. We assume that there will be large differences even within a single tumor or in pre-cancerous tissue (e.g., dysplasia, leukoplakia); and we speculate that there are cells characterized by the impact of a single or of only two affected category that may perhaps explain the *restitutio ad integrum* in some cases, or the reverse of malignancy after transplantation of non-cancerous mitochondria into cancer cells (see Introduction). In these transitions, epigenetics must play an important role to stabilize the metastable state and the progressive adaptation process. However, Bartesaghi and colleagues (Bartesaghi et al., 2015) were able to show that inhibition of mitochondrial metabolism leads to p53 genetic inactivation; this gives unidentified insights into the relationships between mitochondria, genomic stability, and tumor-suppressive control mechanisms. These data are a good example of a causal link between the perturbation of one category in this case energy—and the ensuing perturbation of a second one—information—that finally leads to cancer cell formation. Two questions in the context of our hypothesis are interesting. (A): Are there further mechanisms that become affected in that model because mitochondrial disturbance must initially be associated with a loss of energy that must affect other mechanisms and reactions too, and (B): What is the role of the environment (cell culture conditions) in that process? We suppose that artificial cell culture conditions also contribute to the results obtained.

Third, for cancer cell formation, the cell must preserve the capability to proliferate. If that ability becomes lost, then the cell is not able to adapt through an evolutionary process and cannot adjust the mechanisms it uses to preserve life. It is generally accepted that a normal cell has various different options to initiate and to preserve proliferation (Alberts et al., 2011), but in cancer cells the situation is different and the exact mechanisms why cancer cells show such a various and uncontrolled proliferation is not clear. For example, Hanahan and Weinberg (2011) discussed in their review that the induction of proliferation in cancer cannot be described simply by excessively elevated signaling by oncoproteins such as RAS, MYC or RAF. Such elevated signaling can, in fact, provoke a counteracting response from cells, specifically the induction of senescence and/or apoptosis. We suppose that oncogenes play an important role, but we believe that molecular and genetic deterministic descriptions are not sufficient to provide a comprehensive explanation of the induction of proliferation. As we discussed above, the local microenvironment is important for protein structure and function, too, and such modifications must have impact in the proliferation activation/inhibition cascade. Furthermore, the most processes and proteins depend on sufficient ATP, GTP, and other energy bearing molecules (energy) supply, and if the energy concentration becomes too low, then they lose activity including processes which are involved in proliferation mechanisms. Moreover, Szent-Györgyi (1977) suggested many years ago that disorder produced by practically any influence has the ability to induce proliferation. He described, in a comprehensive model, that a small molecule such as methylglyoxal probably plays an important role in that process. However, the capability to proliferate undoubtedly underpins cell transformation and cancer development.

Furthermore, we suggest: cells that persist in a metastable state without proliferation have already been described as senescent cells (Childs et al., 2015).

## Information, entropy and disorder

“Information is the difference that makes a difference”

Gregory Bateson

**Figure 3:**
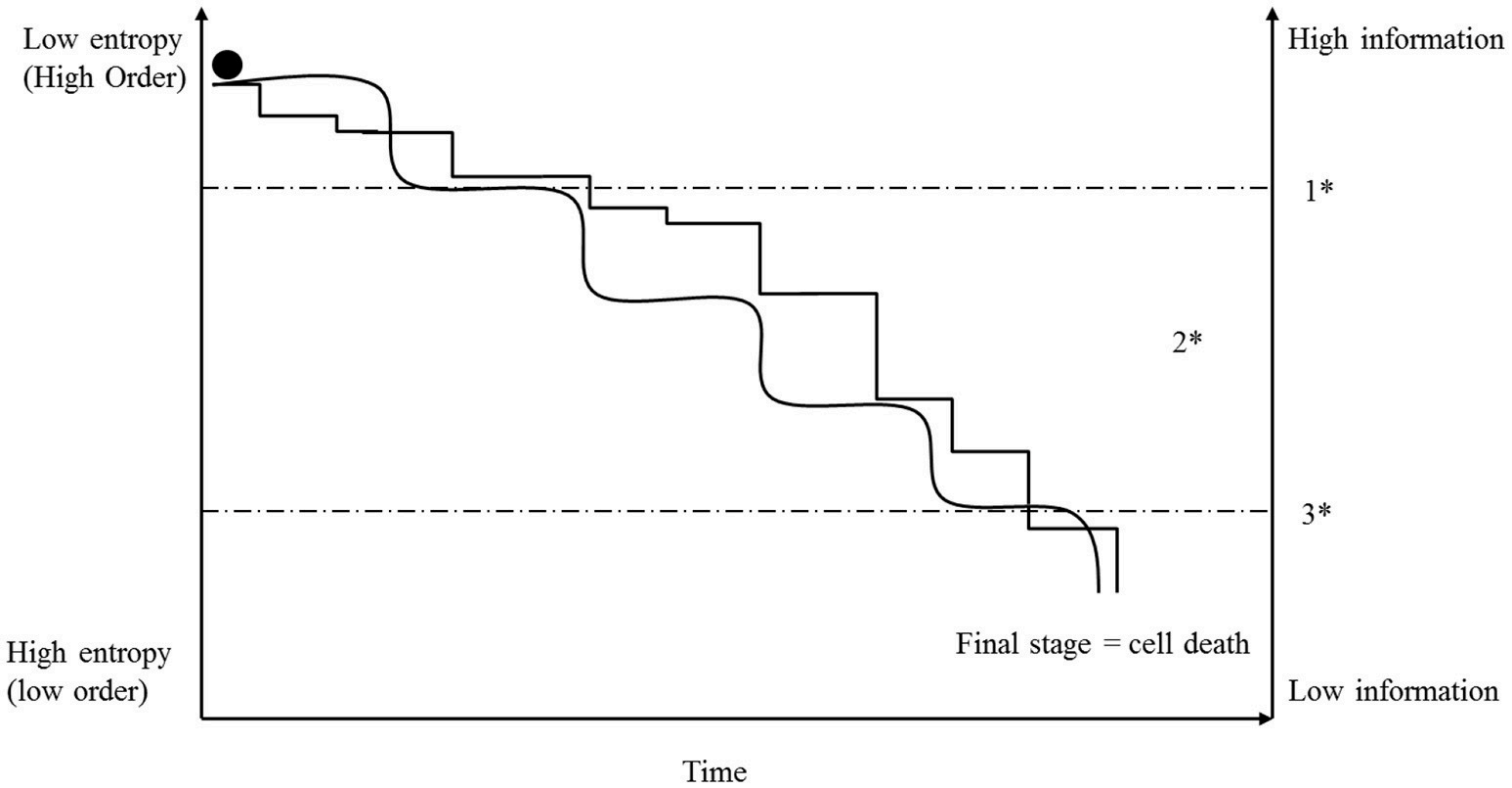
**Initially, a cell possesses a high order and information level (low entropy)**. Because of replication mistakes or for other intra- or extra-cellular reasons, mutations take place and entropy of information accumulates step-by-step (angled line). The consequence of a mutation depends on the importance of the affected (nucleotide) sequence and is symbolized in the graph by the different heights of the steps. In contrast, aneuploidy and CIN can affect several hundred genes in one event and for that reason, accumulation of entropy can increase in larger steps (rolling steps). However, once entropy of information exceeds a specific breaking point (1^*^), so many initially ordered mechanisms are irreversibly disturbed that the cell transitions into a metastable state (2^*^). In that state, the fate of the cell depends on its ability to achieve an acceptable stability in accordance with available energy and matter (see text). Many cells exceed the breaking point (3^*^) and die, but a few will be able to establish a metastable order based on a lower information content and may display all the characteristics of a cancer cell (see Figure 2). In that context, it is very surprising how many mutations and chromosomal aberrations cancer cells can tolerate and survive.

Defining the basic principles of molecular information formation and the mechanisms for the generation of intrinsic information in molecules is highly complex and is based on fundamental physical laws (Umpleby, 2007; Davies et al., 2013; Tarabichi et al., 2013; Wolfe, 2015). However, in the context of this paper, information is defined as molecules (e.g., DNA, RNAs and proteins) which are transmitters and/or recipients of information with different information contents. Every molecule bears its own intrinsic information, which makes it unique, and any change of its composition or energy load will modify the information content, which could lead, for example, to changes of reactivity, molecule structure, molecular code or molecular disintegration. Based on their intrinsic information, myriads of molecules develop and establish morphological structures, activation or inhibition cascades, and all other cell functions and mechanisms by self-organizing processes (Karsenti, 2008; Frieden and Gatenby, 2011; Saetzler et al., 2011). Because of its crucial importance for life, information, especially genes, stands at the focal point of biology and cancer research, and in recent decades some hypotheses have been formulated that indicate that specific changes in genes are the cause of cancer development. In fact, some cancer diseases, such as retinoblastoma and hereditary forms of breast cancer, are based on particular gene mutations (Lohmann and Gallie, 2015; Kleibl and Kristensen, 2016). However, at the same time, most scientists believe that information disturbances achieved during a lifetime are solely responsible for non-hereditary cancer diseases; and there are manifold ways of changing or disturbing cellular information. There is a general acceptance that specific mutations in cancer-associated genes are sufficient to induce processes that finally establish a somatic cancer cell (Hanahan and Weinberg, 2011; Tomasetti and Vogelstein, 2015). Others maintain that unspecific, large chromosomal rearrangements (aneuploidy) are the first step in initiating a progressive increase of disorder that can lead to cancer development (Boveri, 1914; Storchova and Kuffer, 2008; Duesberg et al., 2011). In fact, both mechanisms belong to the same fundamental category—information. Therefore, we suggest that any type of information disturbance can induce cancer by changing and influencing molecular mechanisms described by the state function, entropy (see Figure 3). However, the difference between the two hypotheses is that the accumulation of mutations in a cell is a slow step-by-step process, whereas chromosomal rearrangements can influence several 100 genes in one single event (Breivik, 2005). A consequence of this difference for our hypothesis is that structural or functional information exceeds the breaking point of irreversibility in chromosomally imbalanced cells faster than in mutated ones (see Figure 3). Once that breaking point is passed, the cell is confronted with an irreversible disruption of formerly ordered structures and functions, and the future of the cell depends on the degree and type of the disorder state that is established (see above: metastability). The cell is now situated in the already-mentioned process of fatal interplay of the three fundamental categories (see above) and its future will depend on survivability with a “minimum information” content (Frieden and Gatenby, 2011) and appropriate adaptations of the other two categories (matter and energy).

**Figure 4:**
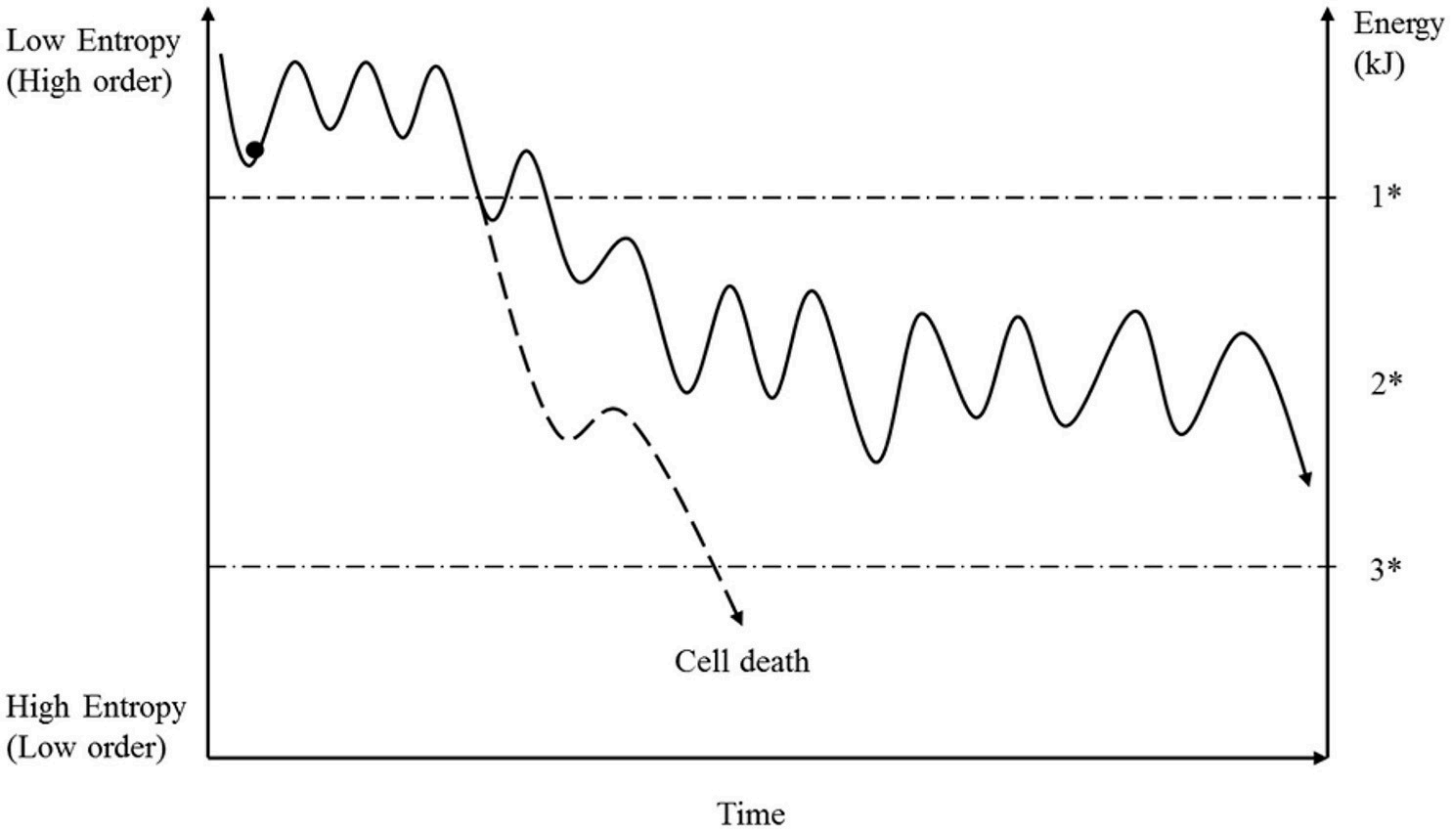
**Under normal conditions a cell permanently ingests fuel and produces energy molecules such as ATP by respiration**. For various reasons, the energy concentration under physiological conditions is not stable (see text) resulting in a reversible fluctuation of cellular entropy. As long as the energy concentration does not fall below a specific level, then all cellular mechanisms will run properly. But if the energy deficit exceeds that value (1^*^), then all cellular mechanisms will be affected, to a greater or lesser extent, and the initially ordered systems will transition into a metastable state (2^*^). The future of the cell now depends on the degree of the energy loss and the particular details of the systems affected. Based on these conditions, the cell has to achieve a new stable state, depending on the availability of matter and information. Because energy loss has an immediate influence on matter transportation and the preservation of information, it is difficult for the cell to establish a stable state (see Figure 2). Moreover, if the energy concentration is too low, then the cell dies (3^*^).

There is one important aspect that we have to consider when we compare the progression of entropy development induced by information with that induced by energy disturbance, in an open cellular system. Information disturbance by mutations or aneuploidy is an accumulative process (see Figure 3) that, in the case of mutations, is limited by cellular repair mechanisms (Iyama and Wilson, 2013). For that reason, entropy increases more or less constantly. On the other hand, a cell possesses, in part, the ability to compensate for an acute loss of energy, and this compensation results in a fluctuation of local energy concentration and intracellular entropy (see Figures 2, 4). This aspect has to be considered when assessing the interplay of these categories (information and energy) during the cancer-development process. In this context, the basis on which matter and environment affect cells differs from the basis on which information and energy exert their effects. Whereas, information and energy are both closely linked to the cell, matter and environment are defined by other cells too, and they depend upon external factors such as nutrition, perfusion, and morphology as well as the entropy released by surrounding cells, which is the subject of one of the next sections.

## Energy and entropy

The second law of thermodynamics states that within a thermodynamically closed system atoms and molecules tend toward a maximum of disorder, whereas energy gives systems the ability to organize themselves in dynamic patterns and structures. Cells as thermodynamically open, ordered and self-organizing systems highly depend on a sufficient energy and matter supply (Molnar et al., 2011; Saetzler et al., 2011; Davies et al., 2013; Tarabichi et al., 2013; Gomez, 2014).

Otto Warburg and coworkers described for the first time that cancer cells metabolize a high amount of glucose to lactic acid, even under normoxic conditions. This phenomenon is well known as aerobic glycolysis or the “Warburg effect.” Yet Warburg's hypothesis includes much more than aerobic glycolysis. He also formulated that cancer results from irreversible mitochondrial (grana) dysfunction and an initial loss of energy induced by hypoxia, toxic agents, radiation or other reasons that later become compensated by glycolysis, or by other mechanisms such as glutaminolysis (Warburg, 1924, 1956; Keenan and Chi, 2015). Moreover, he distinguished between two forms of energy loss: first, the already-mentioned demonstration that cancer cells show an irreversible loss of ATP production by respiration; and second, the “morphological inferiority” of glycolytically synthesized ATP (Warburg, 1924, 1956):

“…One would think that it is immaterial to the cells whether they obtain their energy from respiration or from fermentation (glycolysis), since the energy of both reactions is transformed into energy of adenosine triphosphate…This equation is certainly correct chemically and energetically, but it is incorrect morphologically, because, although respiration takes place for the most part in the structure of the grana (mitochondria), the fermentation enzymes are found for a greater part in the fluid protoplasma (cytosol). The adenosine triphosphate synthesized by respiration therefore involves more structure than the adenosine triphosphate synthesized by fermentation.” Otto Warburg (1956).

His suggestion that energy shows a variable distribution pattern is important and we have to take this aspect into account because, for example, several studies have suggested that enzymes can become differentially activated by energy gradients and that those gradients play an important role during mitosis (Bastiaens et al., 2006; Karsenti, 2008; Fuller, 2010). Furthermore, it is well known that mitochondria are linked to the interphase microtubule network. This link is necessary because cells transfer mitochondria to areas where a higher energy concentration is needed. For example, Lawrence and Mandato were able to show that, after anaphase onset, mitochondria are recruited toward the site of cleavage furrow formation, where they remain enriched as the furrow ingresses until completion of cytokinesis (Lawrence and Mandato, 2013). Aw was able to show that the mitochondrial distribution profiles in jejunal enterocytes changes from basal to apical while rats undergo fasting (Aw, 2000). Furthermore, he describes ATP and oxygen gradients under hypoxic condition (Aw, 2000; Aw and Jones, 1985). We therefore assume that changes from energy gradients to amorphous distributed energy must have consequences for cancer cell formation too. For example, an optimal local concentration with tubulin alpha but without locally sufficient energy (GTP) during mitosis is deleterious because of the strong reciprocal dependency of both molecules in microtubule catastrophe and rescue mechanisms (Alberts et al., 2011; Gardner et al., 2013; Brouhard, 2015). We believe that even a temporary energy loss can provoke chromosomal imbalance and aneuploidy. Furthermore, we suggest that any relevant energy loss can have a major impact in provoking irreversible changes in other cellular functions, such as in DNA replication and repair, in the induction of apoptosis, and in others not yet characterized.

In his publication, Warburg (1956) discussed the relevance of initial energy loss (ATP) but, apart from his assumption that energy deprivation has three aspects (initial loss, energy compensation and morphological inferiority), and his remark that these changes provoke changes leading to cancer development, he did not explain which disturbed mechanisms support this process.

Cells have to synthesize their own usable energetic molecules (e.g., ATP, GTP) from ingested matter (e.g., glucose and oxygen) mainly by respiration, and the distribution of energy is influenced by several factors: (1) Morphological: for example, ATP creates concentration gradients around mitochondria and the diffusion behavior depends on surrounding structures (Aw, 2000; Bastiaens et al., 2006; Karsenti, 2008; Fuller, 2010). (2) Metabolic: because of increased metabolic requirements, for example, due to higher biosynthesis rates or to stress responses, cells accelerate their energy consumption, which impacts upon energy flow. (3) Biologically, because the energy demands have to be adapted during cell cycle and mitosis (Karsenti and Vernos, 2001; Fuller, 2010; Alberts et al., 2011). Based on that assumption, we can postulate that energy availability under physiological conditions shows a high fluctuation rate, both locally and generally (see Figure 4). As long as the cell is able to provide sufficient energy, then the molecular mechanisms described by entropy fluctuate within a range that is compatible with physiological cell functions and structures. But if the energy content decreases even temporarily below a certain concentration level, then some molecular mechanisms shift from a reversibly fluctuating state to irreversibility. This shift is associated with an increase of entropy. Once the cells have entered this state, all energy-dependent systems must suffer under this energy deficiency, to a greater or lesser extent, with consequences for cell structure and functionality. The replication, repair and mitotic processes must, in particular, be significantly affected, which leads to an increased rate of mutations and aneuploidy, as has already been shown by Bartesaghi et al. (2015). However, because the cell has, in such situations, limited opportunities to immediately compensate for energy loss, it has limited opportunities to overcome this critical situation. Its destiny is decided by the degree of energy loss, the ability to ingest matter that is needed and the provision and availability of locally requested information. Reestablishing the original state is not possible, so the cell has to find a new order state through an evolutionary process by fluctuating on a higher entropy state level (see Figure 2). This state we define as a metastable state and as long as the cell maintains the capacity for self-preservation and reproduction then there is a risk that cancer cells are generated.

## Matter, cell environment and entropy

A cell as an enveloped but open system is highly dependent upon an environment that provides all matter, within a defined range of concentrations, which is needed to sustain self-preservation and reproduction. To guarantee an optimal supply, the cell is surrounded by an extracellular matrix that contains all molecules [e.g., growth factors, nutrients, electrolytes, oxygen, protons (defined pH)] required and that provides conditions that are optimized to the appropriate functions of the cell. Based on its specific environment and genetic setting, any cell develops its own characteristics and adapts as one part of a functional multi-cell unit; bronchial tissue is an example (Alberts et al., 2011). But what happens if this sophisticated adjustment is disturbed? What happen if the surrounding matter changes significantly or the cell becomes confronted with toxic agents? To answer these questions, two factors have to be considered, the type and the extent of the perturbing factors.

There are many ways of altering the cellular environment in a negative way, and in the past many experiments have been carried out that clearly show that cells exposed to a non-physiological environment such as toxic agents (Tsutsui et al., 2000; Tayama et al., 2008), inflammation (Kamp et al., 2011; Marusawa and Jenkins, 2014), low pH (Takeshi, 1995; Gatenby and Gillies, 2008), hypoxia (Fang et al., 2008), infection (Castello et al., 2010), irradiation (Nishisgori, 2015), and others can induce significant genetic, metabolic, morphological and cell behavior changes and can initiate cancer development. But do all these factors exert their effect only by influencing genes that finally lead to cancer formation, as the supporters of the mutation theory predict? We claim that qualitative and quantitative changes in environmental matter can also negatively influence energy production, e.g., mitochondrial disturbance by heavy metals (Meyer et al., 2013) or hypoxia (Seyfried, 2015), and that these can have the same consequences as already described in the chapter above. It is clear that the close interaction and interdependency of the three fundamental categories of matter, energy and information are crucial for life and are equally responsible for the initiation and progression of cancer.

## Conclusion

The hypothesis of cancer development suggested here proposes a change in the view of cancer development from a biologically mechanistic process, represented, for example, by genes that determine cell transformation through a linear chain of causal events, to a physical, biochemical, biological-dynamic and, to a certain extent, stochastic, process. To contend that (only) mutations in cancer-relevant genes are exclusively able to induce a complex disease such as cancer underplays the essential complexity of life and excludes the two other fundamental physical categories energy and matter—as causes that are equally responsible for cancer initiation and progression.

Entropy is difficult to pin down, especially in the context of life and cellular systems, but we are convinced that the acceptance of that functional state as a dimension to describe, and to make statements about, the mechanisms responsible for cancer initiation and progression is of great importance.

## Author contributions

RH and CW contributed equally to this work and share first authorship.

### Conflict of interest statement

The authors declare that the research was conducted in the absence of any commercial or financial relationships that could be construed as a potential conflict of interest.
